# Multivariate Analysis of Risk Factors for In-Hospital Dislocation Following Primary Total Hip Arthroplasty

**DOI:** 10.3390/jcm13123456

**Published:** 2024-06-13

**Authors:** Hunter B. Jones, Andrew J. Hinkle, Yida Liu, Senthil N. Sambandam

**Affiliations:** 1 Department of Orthopedic Surgery, University of Texas Southwestern Medical Center, 5323 Harry Hines Blvd, Dallas, TX 75390, USA; andrew.hinkle@utsouthwestern.edu (A.J.H.); yida.liu@utsouthwestern.edu (Y.L.); senthil.sambandam@utsouthwestern.edu (S.N.S.); 2 Department of Orthopedic Surgery, VA North Texas Health Care System, Dallas, TX 75216, USA

**Keywords:** total hip arthroplasty, periprosthetic dislocation, hip instability

## Abstract

**Background:** Early dislocation following primary total hip arthroplasty (THA) is a rare but devastating complication and represents a source of patient morbidity and financial burden to the healthcare system. The objective of this study was to identify patient characteristics and comorbidities that are associated with increased early in-hospital dislocation rates following primary THA. **Methods:** A retrospective cohort study was conducted using patient data from the Nationwide Inpatient Sample (NIS) database; we identified patients who had undergone THA from 2016 to 2019 and compared those with an early periprosthetic dislocation prior to discharge to those without. The patient characteristics and comorbidities were compared using univariate analysis with a subsequent investigation of statistically significant variables using multivariate analysis. The variables were compared using chi square, Fisher’s exact test, and independent sample t-tests with data assessed using odds ratio with 95% confidence intervals. **Results:** A total of 5151 patients sustained an early dislocation compared to 362,743 who did not. Those who sustained an in-hospital dislocation were more likely to share the following characteristics: female sex (OR 1.21, *p* < 0.01), age > 70 (OR 1.45, *p* < 0.01), Caucasian ethnicity (OR 1.22, *p* < 0.01), SLE (OR 1.87, *p* < 0.01), and Parkinson’s disease (OR 1.93, *p* < 0.01). Certain characteristics were also associated with decreased odds of having an in-hospital dislocation including elective surgery (OR 0.14, *p* < 0.01), tobacco use (OR 0.8, *p* < 0.01), diabetes without complications (OR 0.87, *p* < 0.01), and a history of heart valve replacement (OR 0.81, *p* < 0.01). The length of stay was significantly longer (4.7 days vs. 2.3 days) as was the total hospital charges (USD $101,517 vs. USD $66,388) for the early in-hospital dislocation group. **Conclusions:** Several patient characteristics and comorbidities are associated with early in-hospital dislocation episodes following total hip arthroplasty including female sex, age > 70, non-elective surgery, SLE, and Parkinson’s. This information may be useful to help guide intraoperative implant selection and/or postoperative protocol in select patient populations to limit early instability as well as decrease the financial burden associated with this postoperative complication.

## 1. Introduction

Total hip arthroplasty (THA) is a highly effective surgery for patients with degenerative hip joints or femoral neck fractures. Furthermore, it has an excellent track record, leading some to refer to it as the operation of the twentieth century [1]. It has been shown that the annual volume of THAs has increased by close to 177% over the past 20 years. Future projections estimate that 700,000 of these procedures will be performed by the year 2040 in the United States alone [2].

While it was previously understood that aseptic loosening was the most common reason for revision THA (rTHA) [3], recent data suggest that instability has become a more common indication, ranging from 17 to 22% of all revisions [4,5]. Most of these dislocations occur early, within 6 months. Some of the literature suggests that instability within the first two years is responsible for nearly three times as many rTHA procedures as compared to after two years [6].

THA instability is caused by multiple factors that generally relate to patient characteristics, surgical details, and/or postoperative management. Classically, age has been one of the more important patient-related factors, with an increased risk of dislocation at ages over 80 [7,8]. While female sex was previously thought to be a risk factor, the more recent literature suggests this is not the case [9]. Instability is also influenced by the presence of nervous system disorders such as Parkinson’s disease [10]. Although different surgical approaches have been considered to result in varying dislocation rates [11], this is generally less agreed upon in the current literature. Surgeon experience along with prosthesis selection/design are two other surgery-related factors that play a role. Lastly, postoperative management is also important for guiding patients in the recovery period after THA. Modalities such as high-risk-position avoidance, assistive devices, and hip abduction orthoses are all important to consider.

The average length of stay following THA reported in database studies is around 2.97 days [12], and there is a small but important subset of patients who sustain in-hospital dislocation in the immediate postoperative period. However, there are very little data examining the incidence during this time period along with the associated financial burden that it imposes on the medical system.

The objective of this study was to identify patient characteristics and comorbidities that are associated with early in-hospital dislocation rates following primary THA.

## 2. Methods

We utilized a retrospective analysis of the Nationwide Inpatient Sample (NIS) database. We then queried for all patients who underwent primary THA from 2016 to 2019 using International Classification of Disease, Tenth Revision (ICD-10) codes (Table A1). We further stratified this population into two cohorts based on the presence or absence of an in-hospital dislocation following THA. The NIS strictly contains inpatient information only; therefore, data were included from index procedure admission to discharge. Patient demographics such as age, sex, and race were obtained along with length of stay, total charges, and disposition at discharge (routine, short-term hospital stay, alternate facility, death, etc). Select medical comorbidities were also obtained. This study was exempt from IRB approval since the data are publicly available and lack identifying information. To further protect against patient confidentiality, patient values between 1 and 10 were not reported per the healthcare cost and utilization project data agreement.

We used SPSS software version 27.0 (IBM, Armonk, NY, USA) for our statistical analysis. Continuous variables were described using mean value and analyzed with the two-sided independent sample *t*-test. Categorical variables were described using frequency and analyzed using the chi-square test, although the Fisher’s exact test was used for values less than five. Additionally, we utilized multivariate analysis (MVA) for variables that came to show statistically significant associations on univariate analysis (UVA). We calculated the odds ratio (OR) and 95% confidence intervals to assess our variables. We used a *p* value of 0.05 to define significance.

## 3. Results

The NIS database identified 367,894 patients who underwent primary THA between 2016 to 2019. Of these, 5151 patients (1.4%) were reported to have sustained an in-hospital dislocation during the same admission.

The average age of those with a dislocation was 68.2 years versus 65.8 years for those without dislocation. Patients in our dislocation cohort had a higher incidence of age over 70 (47.5% vs. 38.4%) (OR 1.45, *p* < 0.01). Patients with a dislocation were more likely to be female (63.2% versus 55.8%) (OR 1.36, *p* < 0.01). Caucasians made up 87.7% of the dislocation group compared to 85.5% of the non-dislocation group (OR 1.2, 95% CI 1.09 to 1.38, *p* < 0.01) while African Americans made up 6.1% of the dislocation group compared to 7.8% of the non-dislocation group (OR 0.76, *p* < 0.01). There was not a significant difference between the remainder of the different ethnicities (Table 1).

Only 60.3% of the dislocation group underwent THA as an elective procedure with the rest being non-elective for trauma or other medical indications. This is in comparison to the non-dislocation group with 91.8% being elective procedures (OR 0.14, *p* < 0.01). Patients in the dislocation group were at a significant risk of non-home discharge (Table 2). Furthermore, 0.45% of patients in the dislocation group died during admission compared to 0.09% of the non-dislocation group (OR 5.7, *p* < 0.01). The average length of stay for those with a dislocation was 4.7 days compared to 2.3 days, while the average total hospital charges were USD 101,517 compared to USD 66,388, respectively (Table 2).

Univariate analysis showed that patients in the dislocation group had an increased incidence of CKD (7.6% vs. 5.9%, *p* < 0.01), requirement of dialysis (0.2% vs. 0.1%, *p* < 0.01), systemic lupus erythematosus (SLE) (0.9% vs. 0.5%, *p* < 0.01), Parkinson’s disease (1.7% vs. 0.5%, *p* < 0.01), cirrhosis (0.5% vs. 0.3%, *p* < 0.01), history of organ transplant (0.4% vs. 0.2%, *p* = 0.01), presence of pacemaker (2.4% vs. 1.5%, *p* < 0.01), and presence of colostomy (0.2% vs. 0.1%, *p* = 0.02). These patients were also shown to have a decreased incidence of tobacco use (12.2% vs. 17.4%, *p* < 0.01), obesity (19.3% vs. 21.8%, *p* < 0.01), and diabetes (DM) without complications (8.6% vs. 10.0%) (Table 3).

After multivariate analysis, we found that those who sustained an in-hospital dislocation were more likely to share the following characteristics: female sex (OR 1.21, *p* < 0.01), Caucasian ethnicity (OR 1.22, *p* < 0.01), SLE (OR 1.87, *p* < 0.01), and Parkinson’s disease (OR 1.93, *p* < 0.01). Certain characteristics were also associated with decreased odds of having an in-hospital dislocation including elective surgery (OR 0.14, *p* < 0.01), tobacco use (OR 0.8, *p* < 0.01), diabetes without complications (OR 0.87, *p* < 0.01), and history of heart valve replacement (OR 0.81, *p* < 0.01) (Table 3 and Table 4).

## 4. Discussion

Our incidence of 1.4% of patients who sustained an in-hospital dislocation following THA was unexpectedly high. A multivariate analysis assessing the risk of dislocation in a Charnley hip replacement by Berry et al. reported a 1% risk at 1 month postoperatively with an approximate 1% increase in risk per year thereafter [13]. In another database study by Gausden et al., it was shown that 1.4% of THA patients had a readmission within 6 months relating to instability [14]. However, their rate is slightly lower than another Medicare database study by Goel et al., who reported a rate of 2.14% [15]. An international study out of the Danish Hip Arthroplasty Registry by Hermansen et al. showed a two-year cumulative incidence of dislocation to range from 2.2% to 4.3%; however, there was significant hospital variation depending on volume [16]. Nevertheless, our incidence of 1.4% of in-hospital dislocation further emphasizes the significance of this issue. No studies specifically looked at rates of dislocation in the immediate postoperative period while still admitted.

Our study was able to highlight several factors that appear to increase the odds of sustaining an early dislocation in the immediate postoperative period, notably older age, female sex, non-elective surgery, SLE, and Parkinson’s disease. This is the first study to our knowledge that has evaluated these risk factors specifically in the immediate postoperative period.

Older age has previously been understood to be a risk factor for instability following THA [17,18]. We found an OR of 1.45 in patients over the age of 70. This is in agreement with Berry et al., who noted a relative risk of 1.3 for dislocation in patients over the age of 70 [13]. Some older studies have cited dislocation rates upwards of two to three times higher in patients over the age of 80 [7,8]. However, Gausden et al. did not find a correlation between age and an increased risk for dislocation [14]. Our results would suggest that this is not the case, particularly in the setting of an early in-hospital dislocation with the etiology speculated to be related to poorer tissue quality and a decreased muscular envelope.

The literature regarding female sex as a risk factor has been less agreed upon. Females had previously been reported to be at increased risk of dislocation compared to males, with some studies citing ratios of up to 3:1 [13,19]. However, other studies disagree with this assertion [20]. Our study cites an OR of 1.21, which is in agreement with the majority of the literature on the matter, furthering the hypothesis that there may be a difference in soft tissue laxity and/or postoperative range of motion as the root cause [14].

THA for displaced femoral neck fractures (DFNFs) has also been understood to be a risk factor for instability. In our study, THA procedures were reported as either elective or non-elective procedures, with the assumption that non-elective surgery was performed secondary to DFNFs in most instances. Our results suggest that elective surgery confers 86% less likelihood of sustaining an in-hospital dislocation when compared to surgery for a DFNF. This is in agreement with the literature with the reported overall dislocation rates ranging from 6% to 20%, significantly higher than primary THA for osteoarthritis (OA) [17,21].

Our data imply that Parkinson’s patients have a 93% greater odds of sustaining an in-hospital dislocation, which is in agreement with the prior literature with the overall rates of dislocation reported to be as high as 4 to 7% [17]. This likely relates to neuromuscular control and may be especially important in the immediate postoperative period during early mobilization.

SLE also was found to be a significant risk factor for early dislocation in our study. This is in agreement with the literature, which tends to focus on inflammatory arthritis as a whole [17,22]. In a database study by Viswanathan et al., the rate of dislocation in SLE patients was 2.6% compared to 1.4% in non-SLE patients [23], which would agree with our findings, with soft tissue differences once again hypothesized to be the culprit.

Given the elevated risks that these factors impose, it may be prudent to consider these during postoperative management with differing range-of-motion restrictions, use of abduction pillow orthoses, etc. It may also be useful to consider during surgical planning with implant selection (e.g., larger femoral heads, cup positioning, use of dual mobility design, modular components) and/or decision to perform soft tissue repair on patients with several of these major risk factors.

Early dislocation remains a challenging issue and a large financial burden on the healthcare system. We were able to show that an in-hospital dislocation increases the average cost of a THA by nearly USD 34,000 and more than doubles the length of stay. This also does not account for the cost associated with disposition after discharge from the hospital.

We do acknowledge several limitations in our study design. First, we acknowledge our retrospective study design, as well as collecting data from multiple centers, multiple surgeons, and different postoperative protocols. Second, we do acknowledge the limits of the NIS database, which is based on ICD-10 codes and carries the potential to limit data collection. The NIS also does not report on the duration of each procedure, implant selection or positioning, ambulation delays postoperatively, or skill level of the surgeon operating (attending, fellow, resident, etc.); therefore, their potential effects on in-hospital dislocation could not be included in this study. However, our study is strengthened by the comprehensive nature of the NIS database, including a large, national sample size as well as the inclusion of more urgent THA cases.

## 5. Conclusions

With the projected increase in THA volume over the coming decade, there is an increased necessity to identify risk factors for adverse events such as early THA dislocation, particularly in the immediate postoperative period. This study identified older age, female sex, SLE, and Parkinson’s disease as risk factors for early in-hospital dislocation, while elective surgery appeared to decrease risk. These findings can be used as a basis for further research in the field as well as help surgeons implement preventative strategies in these patients who are at high risk irrespective of their experience or technique. Additionally, our findings highlight the financial burden of this problem and can help healthcare policy makers understand the impact that these factors have on healthcare facilities.

## Figures and Tables

**Table 1:** Patient demographics.

	Dislocation Group	Non-Dislocation Group	OR (95% CI)	*p*
Average age at admission	68.2 years	65.8 years		
Age > 70	2445 (47.5%)	139,112 (38.4%)	1.45 (1.37, 153)	**<0.001**
Female sex	3257 (63.2%)	202,485 (55.8%)	1.36 (1.29, 1.44)	**<0.001**
Ethnicity				
Caucasian	4360 (87.7%)	298,742 (85.5%)	1.21 (1.11, 1.32)	**<0.001**
African American	301 (6.1%)	27,261 (7.8%)	0.76 (0.68, 0.86)	**<0.001**
Hispanic	158 3.2%)	12,876 (3.7%)	0.86 (0.73, 1.01)	0.06
Asian	48 (1.0%)	3362 (1.0%)	1.01 (0.76, 1.34)	0.97
Native American	21 (0.4%)	1101 (0.3%)	1.34 (0.87, 2.07)	0.18
Other	82 (1.7%)	5941 (1.7%)	1.0	0.99

**Table 2:** Admission and disposition characteristics.

	Dislocation Group	Non-Dislocation Group	OR (95% CI)	*p*
Elective surgery	3099 (60.3%)	332,454 (91.8%)	0.14 (0.13, 0.14)	**<0.001**
Disposition of patient				
Routine discharge	1296 (25.2%)	141,942 (39.1%)		**<0.001**
Short-term hospital stay	44 (0.9%)	826 (0.23%)		
Intermediate care facility	2137 (41.5%)	65,361 (18.0%)		
Another type of facility	1636 (31.8%)	153,928 (42.5%)		
Home healthcare	12 (0.2%)	241 (0.07%)		
Against medical advice	23 (0.5%)	309 (0.1%)		
Death during admission	23 (0.5%)	309 (0.1%)	5.26 (3.44, 8.05)	**<0.001**
Average length of stay	4.7 days	2.3 days		
Average total charges	USD 101,517.00	USD 66,388.00		

**Table 3:** Patient comorbidities.

	Dislocation Group	Non-Dislocation Group	OR (95% CI)	*p*
Tobacco use	629 (12.2%)	63,079 (17.4%)	0.66 (0.61, 0.72)	**<0.001**
Obesity (BMI 30–40)	993 (19.3%)	78,926 (21.8%)	0.86 (0.80, 0.92)	**<0.001**
Morbid obesity (BMI 40–50)	403 (7.8%)	27,675 (7.6%)	1.03 (0.93, 1.14)	0.6
Super obesity (BMI >50)	31 (0.6%)	1615 (0.5%)	1.35 (0.95, 1.93)	0.09
DM w/o complications	441 (8.6%)	36,387 (10.0%)	0.84 (0.76, 0.93)	**<0.001**
DM w/complications	* (0.2%)	704 (0.2%)	0.90 (0.47, 1.74)	0.75
CKD	391 (7.6%)	21,235 (5.9%)	1.32 (1.19, 1.47)	**<0.001**
Dialysis	12 (0.2%)	371 (0.1%)	2.28 (1.28, 4.06)	**0.004**
HIV	* (0.1%)	497 (0.1%)	0.88 (0.53, 1.43)	0.98
Sickle cell disease	* (0.2%)	646 (0.2%)	0.98 (0.51, 1.90)	1
SLE	44 (0.9%)	1640 (0.5%)	1.9 (1.40, 2.56)	**<0.001**
Parkinson’s disease	89 (1.7%)	1838 (0.5%)	3.45 (2.79, 4.28)	**<0.001**
Ankylosing spondylitis	* (0.1%)	495 (0.1%)	0.85 (0.38, 1.9)	0.85
Cirrhosis	28 (0.5%)	1106 (0.3%)	1.79 (1.23, 2.60)	**0.002**
Hx of transplant	20 (0.4%)	793 (0.2%)	1.78 (1.14, 2.78)	**0.016**
Hx of CABG	143 (2.8%)	8948 (2.5%)	1.13 (0.96, 1.34)	0.16
Hx of PCI	202 (3.9%)	13,062 (3.6%)	1.09 (0.95, 1.26)	0.22
Hx of heart valve replacement	31 (0.6%)	3090 (0.9%)	0.71 (0.49, 1.01)	0.06
Presence of pacemaker	121 (2.4%)	5436 (1.5%)	1.58 (1.32, 1.90)	**<0.001**
Presence of colostomy	12 (0.2%)	433 (0.1%)	1.95 (1.10, 3.47)	**0.02**
Legally blind	* (0.1%)	315 (0.1%)	1.57 (0.74, 3.31)	0.24
Down syndrome	* (0.0%)	128 (0.0%)	0.55 (0.08, 3.94)	0.46

* Numbers between 1 and 10 were not reported per the healthcare cost and utilization project data agreement.

**Table 4:** Multivariate analysis.

	OR (95% CI)	*p*
Female sex	1.21 (1.14, 1.28)	**<0.001**
Caucasian	1.22 (1.09, 1.38)	**<0.001**
African American	0.97 (0.83, 1.14)	0.71
Elective surgery	0.14 (0.14, 0.15)	**<0.001**
Tobacco use	0.80 (0.73, 0.87	**<0.001**
DM w/o complications	0.87 (0.79, 0.96)	**0.008**
CKD	1.05 (0.94, 1.18)	0.37
Dialysis	1.45 (0.80, 2.63)	0.23
SLE	1.87 (1.37, 2.54)	**<0.001**
Parkinson’s disease	1.93 (1.54, 2.43)	**<0.001**
Cirrhosis	1.28 (0.87, 1.88)	0.21
Hx of transplant	1.52 (0.95, 2.41)	0.08
Hx of heart valve replacement	0.57 (0.40, 0.81)	**0.002**
Presence of pacemaker	1.19 (0.98, 1.44)	0.08
Presence of colostomy	1.48 (0.82, 2.65)	0.19

## Data Availability

The original data presented in this study are openly available in the NIS database, which can be found at https://hcup-us.ahrq.gov/db/nation/nis/nisdbdocumentation.jsp, accessed on 6 January 2023.

## Appendix A

**Table A1:** ICD codes used.

THA		Obesity	Comorbidities	Periprosthetic Dislocation
0SR9019	0SRB0JA	E660	Diabetes without complications	T84020A
0SR901A	0SRB0JZ	E6601	E119	T84021A
0SR901Z	0SRB0KZ	E6609		T84022A
0SR9029	0SR90J9	E661	Diabetes with complications	T84023A
0SR902A	0SR90JA	E662	E1169	T84028A
0SR902Z	0SR90JZ	E668		T84029A
0SR9039	0SR90KZ	E669	Tobacco-related disorder	
0SR903A	0SRB019	Z6830	Z87891	
0SR903Z	0SRB01A	Z6831		
0SR9049	0SRB01Z	Z6832		
0SR904A	0SRB029	Z6833		
0SR904Z	0SRB02A	Z6834		
0SR9069	0SRB02Z	Z6835		
0SR906A	0SRB039	Z6836		
0SR906Z	0SRB03A	Z6837		
0SR907Z	0SRB03Z	Z6838		
0SR90EZ	0SRB049	Z6839		
0SRB06Z	0SRB04A			
0SRB07Z	0SRB04Z	Morbid obesity		
0SRB0EZ	0SRB069	Z6841		
0SRB0J9	0SRB06A	Z6842		
		Z6843		
		Z6844		
		Z6845		

## References

[1] Learmonth I.D., Young C., Rorabeck C. (2007). The operation of the century: Total hip replacement. Lancet.

[2] Shichman I., Roof M., Askew N., Nherera L., Rozell J.C., Seyler T.M., Schwarzkopf R. (2023). Projections and Epidemiology of Primary Hip and Knee Arthroplasty in Medicare Patients to 2040–2060. JB JS Open Access.

[3] Soong M., Rubash H.E., Macaulay W. (2004). Dislocation after total hip arthroplasty. J. Am. Acad. Orthop. Surg..

[4] Bozic K.J., Kurtz S.M., Lau E., Ong K., Vail T.P., Berry D.J. (2009). The epidemiology of revision total hip arthroplasty in the United States. J. Bone Jt. Surg. Am..

[5] Gwam C.U., Mistry J.B., Mohamed N.S., Thomas M., Bigart K.C., Mont M.A., Delanois R.E. (2017). Current Epidemiology of Revision Total Hip Arthroplasty in the United States: National Inpatient Sample 2009 to 2013. J. Arthroplast..

[6] Falez F., Papalia M., Favetti F., Panegrossi G., Casella F., Mazzotta G. (2017). Total hip arthroplasty instability in Italy. Int. Orthop..

[7] Pieringer H., Labek G., Auersperg V., Böhler N. (2003). Cementless total hip arthroplasty in patients older than 80 years of age. J. Bone Jt. Surg. Br..

[8] Ekelund A., Rydell N., Nilsson O.S. (1992). Total hip arthroplasty in patients 80 years of age and older. Clin. Orthop. Relat. Res..

[9] Rowan F.E., Benjamin B., Pietrak J.R., Haddad F.S. (2018). Prevention of Dislocation After Total Hip Arthroplasty. J. Arthroplast..

[10] Weber M., Cabanela M.E., Sim F.H., Frassica F.J., Harmsen W.S. (2002). Total hip replacement in patients with Parkinson’s disease. Int. Orthop..

[11] Palan J., Beard D.J., Murray D.W., Andrew J.G., Nolan J. (2009). Which approach for total hip arthroplasty: Anterolateral or posterior?. Clin. Orthop. Relat. Res..

[12] Molloy I.B., Martin B.I., Moschetti W.E., Jevsevar D.S. (2017). Effects of the Length of Stay on the Cost of Total Knee and Total Hip Arthroplasty from 2002 to 2013. J. Bone Jt. Surg. Am..

[13] Berry D.J., von Knoch M., Schleck C.D., Harmsen W.S. (2004). The cumulative long-term risk of dislocation after primary Charnley total hip arthroplasty. J. Bone Jt. Surg. Am..

[14] Gausden E.B., Parhar H.S., Popper J.E., Sculco P.K., Rush B.N.M. (2018). Risk Factors for Early Dislocation Following Primary Elective Total Hip Arthroplasty. J. Arthroplast..

[15] Goel A., Lau E.C., Ong K.L., Berry D.J., Malkani A.L. (2015). Dislocation rates following primary total hip arthroplasty have plateaued in the Medicare population. J. Arthroplast..

[16] Hermansen L.L., Viberg B., Overgaard S. (2022). Large hospital variation in the risk of dislocation after primary total hip arthroplasty for primary osteoarthritis: 31,105 patients in 59 hospitals from the Danish Hip Arthroplasty Register. Acta Orthop..

[17] Meek R.M., Allan D.B., McPhillips G., Kerr L., Howie C.R. (2006). Epidemiology of dislocation after total hip arthroplasty. Clin. Orthop. Relat. Res..

[18] Malkani A.L., Dilworth B., Ong K., Baykal D., Lau E., Mackin T.N., Lee G.C. (2017). High Risk of Readmission in Octogenarians Undergoing Primary Hip Arthroplasty. Clin. Orthop. Relat. Res..

[19] Leichtle U.G., Leichtle C.I., Taslaci F., Reize P., Wünschel M. (2013). Dislocation after total hip arthroplasty: Risk factors and treatment options. Acta Orthop. Traumatol. Turc..

[20] Mahomed N.N., Barrett J.A., Katz J.N., Phillips C.B., Losina E., Lew R.A., Guadagnoli E., Harris W.H., Poss R., Baron J.A. (2003). Rates and outcomes of primary and revision total hip replacement in the United States medicare population. J. Bone Jt. Surg. Am..

[21] Noticewala M., Murtaugh T.S., Danoff J., Cunn G.J., Shah R.P., Geller J. (2018). Has the risk of dislocation after total hip arthroplasty performed for displaced femoral neck fracture improved with modern implants?. J. Clin. Orthop. Trauma..

[22] Ravi B., Escott B., Shah P.S., Jenkinson R., Chahal J., Bogoch E., Kreder H., Hawker G. (2012). A systematic review and meta-analysis comparing complications following total joint arthroplasty for rheumatoid arthritis versus for osteoarthritis. Arthritis Rheum..

[23] Viswanathan V.K., Sakthivelnathan V., Senthil T., Menedal A., Purudappa P.P., Mounasamy V., Sambandam S. (2023). Does systemic lupus erythematosus impact the peri-operative complication rates following primary total knee arthroplasty? A national inpatient sample-based large-scale study. Arch. Orthop. Trauma. Surg..

